# Psychophysiological Responses in Emergency Medical Technician Students during Simulated Work Activities in a Hot Environment

**DOI:** 10.3390/ijerph17103443

**Published:** 2020-05-15

**Authors:** Hayden D. Gerhart, Amy B. Fiorentini, Kristi L. Storti, Robert Alman, Madeline P. Bayles, Louis Pesci, Yongsuk Seo

**Affiliations:** 1 Department of Kinesiology, Health, and Sport Science, Indiana University of Pennsylvania, Indiana, PA 15705, USA; hgerhart@iup.edu (H.D.G.); abftini@gmail.com (A.B.F.); klstorti@iup.edu (K.L.S.); balman@iup.edu (R.A.); mpbayles@iup.edu (M.P.B.); Louis.Pesci@iup.edu (L.P.); 2 Environmental Physiology Laboratory, Kent State University, Kent, OH 44242, USA

**Keywords:** heat stress, simulated activities, selective attention, total mood disturbance

## Abstract

This study compared physiological responses and cognitive performance during simulated work activities in heat to a thermoneutral condition. First responders perform physically demanding activities in a hot environment which may impose additional burdens on tactical personnel during daily tasks. Ten healthy (8 men and 2 women) participants performed two consecutive simulated work activities with two repetitions of each activity (10 min walking on treadmill and 15 sandbag lifts) under heat and thermoneutral conditions. A Stroop color word test (SCWT) and total mood disturbance (TMD) were obtained at first and second baseline (B1, B2), after a 30-min resting period (B3), and recovery (R1). At the end of the trial, core temperature (Tc), skin temperature (tsk), and mean body temperature (Tb) were higher in the heat condition compared to neutral condition (all *p* ≤ 0.05), whereas oxygen uptake, heart rate, and mean arterial pressure were not significantly different between conditions. There were no differences in scores of SCWT and TMD between conditions. However, TMD was significantly improved after two successive bouts of exercise compared to B3 (all *p* ≤ 0.05). This investigation shows that two successive simulated work activities did not induce the detrimental influence on thermoregulatory and cognitive responses. Extended work activities in a hot and humid environment may impose a psychophysiological burden and need to be investigated.

## 1. Introduction

Generally, first responders (paramedics, emergency medical technicians, police officers, firefighters, and rescuers) are involved in the first line of treatment and transportation in emergency situations. The first responders perform strenuous physical activities, such as lifting and carrying of patients and stretchers under different environmental conditions, rarely in comfortable environmental conditions [1]. Working as a first responder often requires various levels of cognitive function to react and make decisions quickly while performing job-related tasks.

Physical activity in the heat is known to significantly decrease exercise capacity [2,3,4] and potentially induce heat exhaustion, heat-related injuries, hyperthermia; body temperature in excess of 40 °C; and possible death [5]. Decrements in aerobic performance have been shown to be independently and concurrently related to increased skin temperature and core temperature, suggesting that the decreased performance is due mainly to cardiovascular strain and altered cardiac and vascular function rather than central nervous system strain, a neurological fatigue which is typically brought on by repeated muscular contraction [2,6].

Adverse events related to heat stress are most frequently observed in a hot environment, but can also be seen in thermoneutral conditions when core temperature reaches a critical point of 40 °C independent of hydration, initial core temperature, rate of heat storage, and acclimation time [5].

Physical activities in the heat increase the rate of metabolism [7], decrease the rate of lactate removal [7], and result in accumulation of blood lactate at a faster rate [8]. The combination of a hot environment and physically demanding activities can lead to excessive dehydration. If dehydration or excessive body fluid loss continues, and fluid is not replaced with water and electrolytes, tissue perfusion can be limited and may induce headaches, fatigue and heat exhaustion [9]. Ultimately, a reduction in body fluid and plasma volume inhibits maintenance of cutaneous circulation and core body temperature, which may result in cardiovascular strain and hyperthermia, or even heat stroke [10,11,12,13]. Dehydration also impairs the subjective perception of exertion, attention, manual dexterity, working memory, and mood state and coordination, all of which result in a high incidence of occupational accidents and injuries [9,14,15,16,17,18] because selective attention, working memory, and cognitive flexibility are considered essential cognitive functions [19].

While physiological responses are negatively affected by hyperthermia and dehydration, special consideration should be given to the influence of physical activities that attempt to simulate first-responder activities in a laboratory setting since there are limited studies examining simulated first-responder activities. Therefore, the purpose of this study was to compare psychophysiological response during simulated first-responder activities in a hot environment compared to a thermoneutral condition. It was hypothesized that psychophysiological responses would be affected by physical activities in a hot environment compared to a thermoneutral condition.

## 2. Materials and Methods

The current study employed a repeated measure, within-subjects design with counterbalanced fashion. Participants reported to the Center for Sport Science Research and Education at Indiana University of Pennsylvania on three occasions (familiarization and two experimental sessions). Two experimental trials (Heat; 37.8 °C dry-bulb, 60% relative humidity [RH] and neutral; 25.0 °C dry-bulb, 40% RH) were separated by at least 3 days, but the trials were conducted at the same time of day. All subjects were not acclimated to the environmental test conditions prior to the trials. The Indiana University of Pennsylvania Institutional Review Board (IRB) approved this study. Informed consent was obtained from all participants before their participation in any part of this study (IRB: 17-205).

### 2.1. Participants

Participants were recruited from the Indiana University of Pennsylvania, Institute for Rural Health and Safety Emergency Medical Technician (EMT) Certification Program. The EMT Certification Program is primarily designed for future emergency medical services (EMS) personnel who may respond to emergencies. In order to be eligible for the study, individuals have to be currently enrolled in the certification program and had to complete a comprehensive health habits, physical activity and behavioral risk factor questionnaire. Individuals were excluded from the study if they reported previous symptoms or diagnosis of cardiovascular, renal, metabolic disease, color blindness, or musculoskeletal injuries. Furthermore, participants were excluded if resting blood pressure exceeded 140/90 mmHg. Based upon these criteria, a total of 10 subjects (8 male and 2 females) (mean ± standard deviation; age 24 ± 2.5 years; height 177.2 ± 4.9 cm; weight 88.2 ± 20.2 kg; and percent body fat 23.9 ± 12.9%) were enrolled in the study.

### 2.2. Procedures

During the familiarization, body weight, height and body composition were assessed using a BodPod (Cosmed, Rome, Italy). Subjects were familiarized with the study procedures and instrumentation, including cognitive function tests, a minimum of three times. Subjects performed a modified Balke treadmill test to determine the maximal oxygen uptake (VO_2_max) utilizing an integrated metabolic measuring system (True Max 2400, ParvoMedics, Sandy, UT, USA), while heart rate (HR) was constantly recorded with a Polar heart rate monitor (Polar RS800 CS, Polar Electro Oy, Kempele, Finland). Thereafter, desired exercise intensity of 75% of HRmax was determined for the experimental trials.

To determine maximal oxygen uptake (VO_2_max), the participants walked at a constant speed of 5.3 km/hr and started at a 0% incline. Each stage increased by 2.5% incline every two minutes until volitional fatigue (corroborated by rating of perceived exertion (RPE) of ≥ 18 [20] or plateau in heart rate or VO_2_). Participants were instructed to ingest a telemetry capsule (CorTemp Temperature Sensor; HQ, Inc.; Palmetto, FL, USA) for measurement of core body temperature (Tc) at least 3 h before arrival to the laboratory as per manufacturers’ instructions since telemetry capsule measurement of core temperature has been proven to be representative of Tc [21,22]. No food or drink was ingested after ingestion of the capsule, as fluid can alter the temperature and food can impact transit time of the capsule.

**Figure 1:**
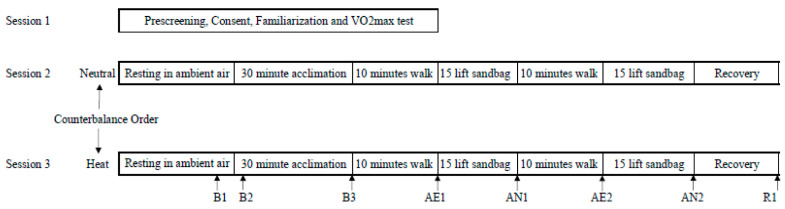
Experimental design and timeline. B1, baseline; B2, second baseline; B3, third baseline, AE1, first walking; AN1, first lifting; AE2; second walking; AN2, second lifting; R1, recovery.

Upon arrival at the laboratory on the day of an experimental trial (Figure 1), participants were asked to void their bladder and provide a urine sample, both before and after exercise, for assessment of urine specific gravity with a refractometer (PAL-10S Pocket Refractometer, Atago, Tokyo, Japan). Participants were given a T-shirt, shorts and athletic shoes upon arrival, and this same attire was used by each subject for both trials. Study instrumentation including heart rate monitor and skin thermistors (ITP082-25, Nikkiso; Therm Co., Ltd., Tokyo, Japan) were affixed with transparent dressing film (Tegaderm, 3M; St. Paul, MN, USA) on the ipsilateral calf, thigh, chest, triceps, and forearm for a calculation of weighted mean skin temperature (Tsk): (0.22 *calf temperature) + (0.28 *thigh temperature) + (0.28 *chest temperature) + (0.14 *forearm temperature) + (0.08 *triceps temperature) [23]. Using Tc and Tsk, mean body temperature (Tb) was calculated as Tb = Tc (0.8) × Tsk (0.2) [24]. Expired air was collected via a mouthpiece with nose clip (Hans Rudolph, KS, USA) and analyzed for VO_2_. Blood pressure (mean arterial pressure [MAP]) was obtained with a standard aneroid sphygmomanometer and stethoscope. Mean arterial pressure was calculated as MAP = (1/3 systolic blood pressure [SBP]) + (2/3 diastolic blood pressure [DBP]).

Following instrumentation with sensors, first baseline measurements (B1) were obtained while participants sat quietly for 15 min in an ambient temperature (25.0 °C, 40% RH). Upon completion of B1, subjects entered the environmental chamber and second baseline measurements (B2) were obtained, followed by a 30-min resting period under a given condition and dependent variables were measured (B3). After the 30-min resting period, subjects performed two consecutive simulated work activities with two repetitions of each activity. The simulated work activities consisted of 10 min walking (AE) and 15 lifts of a 22.7 kg sandbag (AN). The 10 min of walking on the treadmill was performed at an intensity of 70%−75% of their maximum heart rate. If HR exceeded the target HR range for more than one minute, the treadmill speed and incline were adjusted to maintain the same relative intensity for each participant. This adjustment allowed for direct comparisons of psychophysiological alternation between conditions under similar relative intensities of exercise although the workload is absolute in nature. Furthermore, this adjustment allowed subjects to complete the experimental trials and ensure subject safety.

A total of 15 sandbag lifts were performed (22.7 kg, simulating a work activity) from the ground to a table (width 183 cm × height 72 cm) for 5 min, giving the subject 10 s to bring the sandbag to the table and 10 s to place the sandbag back down to the floor. A metronome sounded every second, while the primary investigator provided cues to the subject every 10-s interval for each lift onto and off of the table. Following consultation with the Institute for Rural Health and Safety at Indiana University of Pennsylvania, this protocol was deemed appropriate for this student population. Following two consecutive simulated work activities, subjects stepped out of the environmental chamber and performed 10 min of either a passive or active cool down on a treadmill or cycle ergometer (R1) until HR returned to within 20 bpm of the initial baseline value.

The dependent variables of Tc, Tsk, and HR were recorded continuously throughout the trials and averaged for the last minute of each time point. VO_2_, BP, RPE (6 = no exertion at all, 20 = maximal exertion) [20], thermal sensation (TS; 4 = Very Hot, 0 = Neutral, −4 = Very Cold) [25], and lactic acid (LAC) were recorded during the last 3 min of each time point. The LAC accumulation was assessed through a finger stick with a blood lactate analyzer (Lactate Scout +, EKF Diagnostics, Germany). Upon completion of physiological and subjective measurements, the Stroop color word test (SCWT) and total mood disturbance (TMD) were assessed immediately at B1, B2, B3, and R1 via computerized battery.

### 2.3. Instrumentation

Assessments of SCWT and TMD were conducted using the Automated Neuropsychological Assessment metrics-4th Edition (ANAM4) (Vista Life Sciences, Parker, CO, USA). The SCWT assesses processing speed, selective attention, and executive function. These assessments relate directly to appropriate demands within populations of first responders, who are expected to differentiate task relativity, react quickly, and make life-altering decisions with incredible precision. There are three 45 s tests (word, color, and color-word) used in the SCWT. The first test (word: W) requires the subject to press a corresponding key for each word (i.e., 1 for red, 2 for green, and 3 for blue). Following that they are instructed to press the corresponding key based on color (C). A series of colors including red, green, or blue are presented on the screen. In the final section, a series of words are presented in a color (CW) that did not match the name of the color displayed. The subjects have to press the corresponding key assigned to color. An interference score of SCWT was calculated for each trial as: Interference score = CW − [(W × C) / (W + C)]. A higher score indicates better cognitive performance [26]. The mood state is designed to assess seven categories of mood. These categories include anger, anxiety, depression, fatigue, happiness, restlessness, and vigor. Forty-two (42) words conveying various emotions are displayed on the laptop screen and the subjects were asked to select a number between 0 and 6 describing how they feel. Zero (0) was designated as “Not at All” and 6 was designated as “Very Much”. Later total mood disturbance (TMD) was calculated as TMD = (anger + anxiety + depression + fatigue + restlessness) − (happiness + vigor). A higher TMD score represents poor mood state.

### 2.4. Data Analysis

Statistical analyses were performed using Statistical Package for the Social Sciences 22.0 (IBM-SPSS, Somers, NY, USA). For all analyses, significance was set at an alpha level of <0.05. Using SPSS 22.0, two-way (two condition by eight time points) repeated measures analysis of variance (ANOVA) was performed to evaluate the physiological and subjective measurement between conditions. Another two-way (two conditions by four time points) repeated measures ANOVA was conducted for SCWT and TMD to ascertain any condition effects. When the ANOVA indicated a significant interaction or main effect, a post-hoc pairwise comparison was utilized. Additionally, a paired sample *t*-test was utilized to determine differences between conditions at each time point. The level of statistical significance was set at alpha ≤0.05 and all data are presented as mean ± standard deviation (SD).

## 3. Results

The average VO_2_max and HRmax were 49.7 ± 15.8 ml/kg/min and 187.8 ± 8.1 beats/min, respectively. The corresponding exercise intensity of 75% HRmax was 141.0 ± 6.1 beat/min. Whole body water loss was not significantly different between conditions (*p* = 0.184) with an average of 0.6 ± 0.2 kg in neutral and 0.8 ± 0.4 kg in the heat, inducing an average whole-body water loss of 0.7 ± 0.3 and 0.9 ± 0.5% of initial body weight, respectively.

### 3.1. Thermoregulatory Response

**Figure 2:**
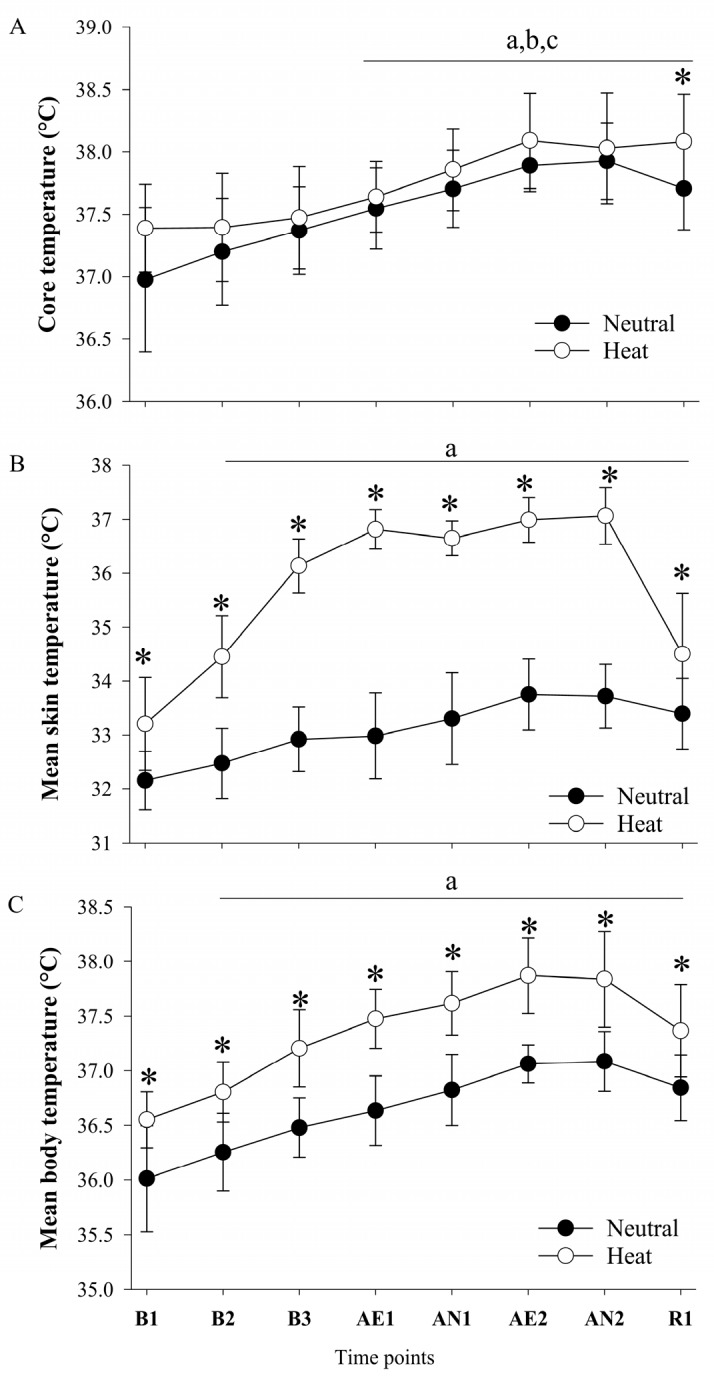
**(A)** Core temperature **(B)** mean skin temperature, and **(C)** mean body temperature response in thermoneutral and heat conditions. B1, baseline; B2, second baseline; B3, third baseline, AE1, first walking; AN1, first lifting; AE2; second walking; AN2, second lifting; R1, recovery. Values are mean ± standard deviation (*n* = 10). ^a^ *p* ≤ 0.05 versus B1; ^b^ *p* ≤ 0.05 versus B2; ^c^ *p* ≤ 0.05 versus B3; * *p* ≤ 0.05 versus heat condition.

There was no significant interaction (F(7,63) = 1.786, *p* = 0.106, $\eta_{p}^{2}$ = 0.166) observed for Tc. However, main effect for condition (F(1,9) = 6.105, *p* = 0.036, $\eta_{p}^{2}$ = 0.404) and time (F(7,63)=28.706, *p* ≤ 0.001, $\eta_{p}^{2}$ = 0.761) indicates that Tc was gradually increased during exercises (AE1 through AN2) and R1 compared to B1 through B3 (all *p* < 0.05) in both conditions (Figure 2A). Tc was significantly higher at R1 in the heat condition (t = 4.444, *p* = 0.002) compared to the thermoneutral condition (Figure 2A). Tsk showed a significant interaction (F(7,63) = 35.014, *p* ≤ 0.001, $\eta_{p}^{2}$ = 0.796), main effect for condition (F(1.9) = 435.216, *p* ≤ 0.001, $\eta_{p}^{2}$ = 0.980), and time (F(7,63) = 82.529, *p* ≤ 0.001, $\eta_{p}^{2}$ = 902), revealing that Tsk progressively increased throughout the trials (all *p* ≤ 0.05), with a significantly greater value from B1 through R1 in the heat condition (all *p* ≤ 0.05) compared to neutral condition (Figure 2B). Tb showed a significant interaction (F(7,63) = 2.987, *p* = 0.009, $\eta_{p}^{2}$ = 0.249), main effect for condition (F(1,9) = 106.040, *p* ≤ 0.001, $\eta_{p}^{2}$ = 0.922), and time (F(7,63) = 62.799, *p* ≤ 0.001, $\eta_{p}^{2}$ = 0.875), indicating that Tb was gradually increased throughout the trials (all *p* ≤ 0.05) compared to baseline 1. Tb was significantly higher at B1 through R1 in the heat condition (all *p* ≤ 0.05) compared to the neutral condition (Figure 2C).

### 3.2. Metabolic and Hemodynamic Response

**Figure 3:**
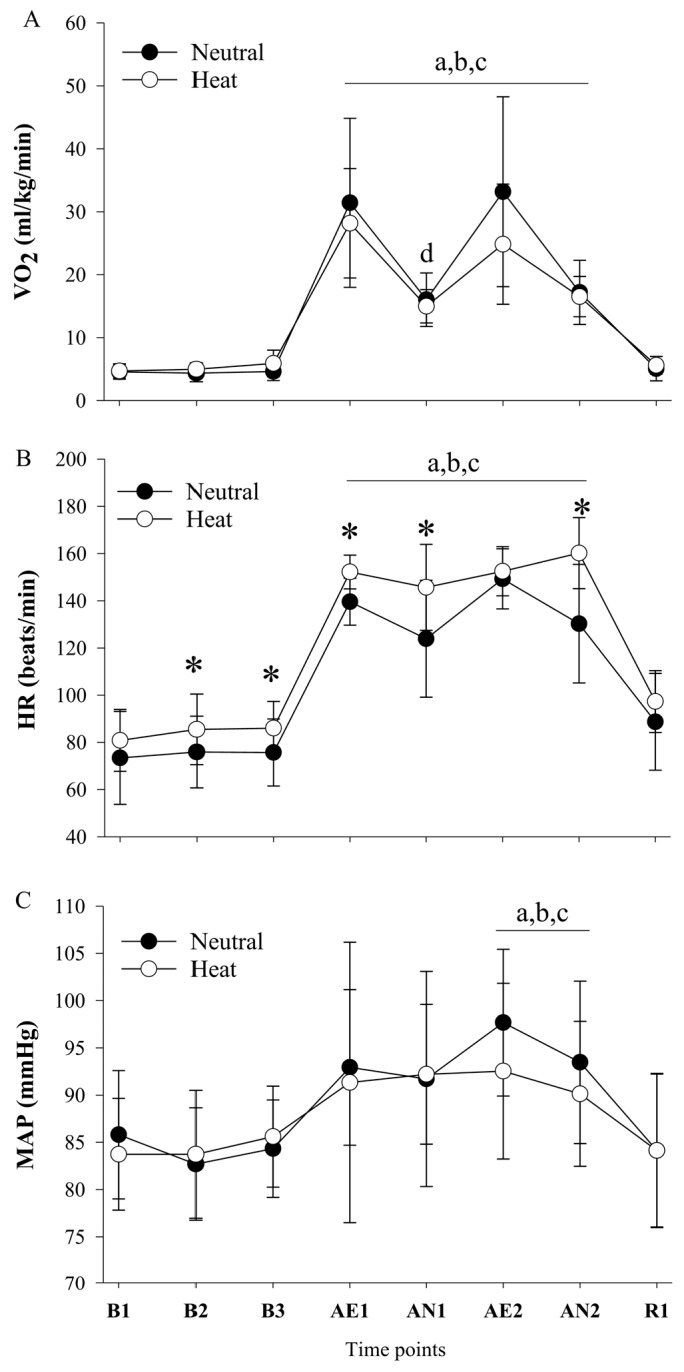
**(A)** Oxygen uptake **(B)** heart rate, and **(C)** mean arterial pressure response in thermoneutral and heat conditions. B1, baseline; B2, second baseline; B3, third baseline, AE1, first walking; AN1, first lifting; AE2; second walking; AN2, second lifting; R1, recovery. Values are mean ± standard deviation (*n* = 10). ^a^ *p* ≤ 0.05 versus B1; ^b^ *p* ≤ 0.05 versus B2; ^c^ *p* ≤ 0.05 versus B3; ^d^ *p* ≤ 0.05 versus AE1; * *p* ≤ 0.05 versus heat condition.

As shown in Figure 3A, VO_2_ indicated a significant interaction (F(7,63) = 5.526, *p* ≤ 0.001, $\eta_{p}^{2}$ = 0.38), but no main effect for condition (F(1,9) = 1.846, *p* = 0.207, $\eta_{p}^{2}$ = 0.17) and main effect for time (F(7,63) = 50.424, *p* ≤ 0.001, $\eta_{p}^{2}$ = 0.849), indicating that VO_2_ was significantly increased during exercise bouts (AE1 through AN2) compared to B1 through B3 (all *p* ≤ 0.05) in both conditions. Interestingly, VO_2_ was significantly decreased during AN1 compared to AE1 (*p* = 0.021). However, there was no significant difference between AE2 and AN2 (*p* = 0.110). As shown in Figure 3B, HR indicated a significant interaction (F(7,63) = 10.904, *p* ≤ 0.001, $\eta_{p}^{2}$ = 0.548), main effect for condition (F(1,9) = 21.651, *p* = 0.01, $\eta_{p}^{2}$ = 0.706), and time (F(7,63) = 140.847, *p* ≤ 0.001, $\eta_{p}^{2}$ = 0.940). HR was significantly increased during exercise bouts (AE1 through AN2) compared to B1 through B3 (all *p* ≤ 0.05). The HR was significantly higher at all-time points in the heat compared to the neutral condition with the exception of B1, AE2, and R1 (all *p* ≤ 0.05). As shown in Figure 3C, MAP indicated no significant interaction (F(7,56) = 1.119, *p* = 0.364, $\eta_{p}^{2}$ = 0.123) and no main effect of condition (F(1,8) = 0.092, *p* = 0.769, $\eta_{p}^{2}$ = 0.011), but main effect for time (F(7,56) = 28.604, *p* ≤ 0.001, $\eta_{p}^{2}$ = 0.781), indicating that MAP was significantly increased during AE2 and AN2 (all *p* < 0.05) compared to B1, B2, and B3.

### 3.3. Blood Lactate Accumulation

**Figure 4:**
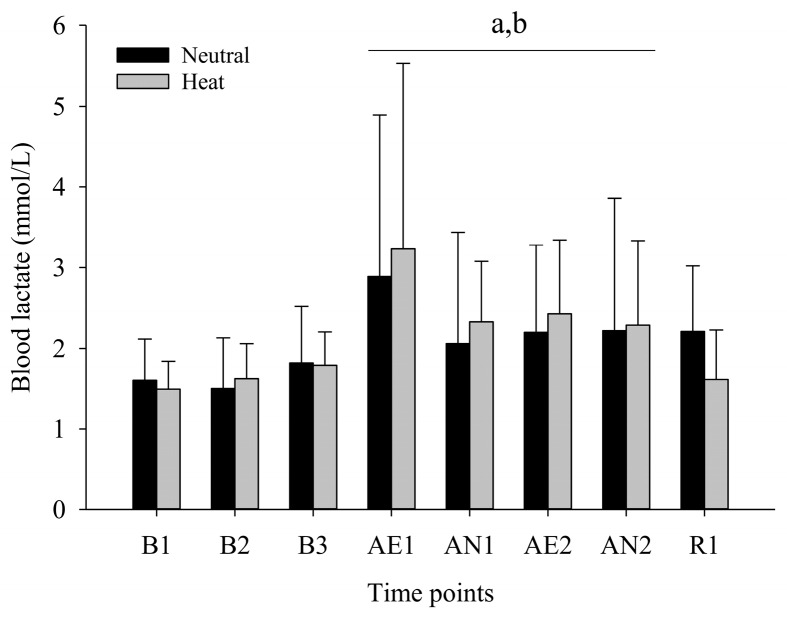
Blood lactate concentration in thermoneutral and heat conditions. B1, baseline; B2, second baseline; B3, third baseline, AE1, first walking; AN1, first lifting; AE2; second walking; AN2, second lifting; R1, recovery Values are mean ± standard deviation (*n* = 10). ^a^ *p* ≤ 0.05 versus B1; ^b^ *p* ≤ 0.05 versus B2.

Blood lactate (BLA) indicated no condition by time interaction (F(7,63) = 0.288 *p* = 0.956, $\eta_{p}^{2}$ = 0.031), main effect for time (F(7,63) = 5.903, *p* ≤ 0.001, $\eta_{p}^{2}$ = 0.396), and no main effect for condition (F(1,9) = 0.077, *p* = 0.787, $\eta_{p}^{2}$ = 0.008), indicating that BLA was significantly increased during exercise bouts (AE1 through AN2) compared to B1 and B2 (all *p* ≤ 0.05) (Figure 4).

### 3.4. Cognitive Performance and Subjective Responses

The interference score of SCWT indicated no significant interaction (F(3,27) = 0.355, *p* = 0.8, $\eta_{p}^{2}$ = 0.036), main effect for condition (F(1,9) = 1.266, *p* = 0.290, $\eta_{p}^{2}$ = 0.123), or time (F(3,27) = 1.644, *p* = 0.203, $\eta_{p}^{2}$ = 0.154). Total mood disturbance (TMD) indicated no significant interaction (F(3,27) = 1.914, *p* = 0.151, $\eta_{p}^{2}$ = 0.175) and no main effect for condition (F(1,9) = 0.649, *p* = 0.441, $\eta_{p}^{2}$ = 0.067), but a main effect for time (F(3,27) = 23.624, *p* ≤ 0.001, $\eta_{p}^{2}$ = 0.724). A pairwise comparison indicated that TMD was significantly impaired at B3 compared to B1 and B2 in both conditions (all *p* ≤ 0.05). TMD was significantly improved at R1 compared to B3 (all *p* ≤ 0.05) (Table 1).

As compared to the thermoneutral condition, RPE was significantly higher during the heat condition at AE2 (t = 3.000, *p* ≤ 0.001), AN2 (t = 4.272, *p* = 0.002). TS was significantly “hotter” during the heat condition at B2 (t = 10.766, *p* ≤ 0.001), B3 (t = 14.697, *p* ≤ 0.001), AE1 (t = 6.708, *p* ≤ 0.001), AN1 (t = 4.993, *p* ≤ 0.001), AE2 (t = 5.582, *p* ≤ 0.001), and AN2 (t = 7.965, *p* ≤ 0.001) (Table 1).

## 4. Discussion

The current study examined the psycho-physiological responses during simulated work activities. We showed that the two simulated work activities elicited similar increases in Tc, independent of environmental condition. This result may be attributed to the short duration of heat exposure and exercise intensity adjustment during the aerobic portion of the protocol. However, we observed a significant increase in Tsk during the heat conditions such that Tb was higher in the heat condition over time (Figure 1). In the current study, the combination of heat stress and two simulated work activities increased Tc about 0.6 ± 0.4 °C and induced mild hyperthermia at 38.0 ± 0.4 °C [27], immediately after the second anaerobic exercise, such that Tc did not increase beyond 1.2 °C. The results showed that selective attention, as measured with SCWT, was not impaired in the heat condition. This result is in agreement with previous studies indicating that cognitive impairment is observed when core temperature increases greater than 1.2 °C from an initial core temperature. This cognitive impairment is attributed to cerebral hypo-perfusion and raised brain temperature, which impairs the neural network of the brain and cognitive processing [19]. Further, dehydration is another factor that is commonly induced by exercise and/or heat stress. A previous study reported that executive function and information processing were impaired when a 2% loss of body water (dehydration) was observed [28,29]. In the current study, the magnitude of whole-body water loss was 0.9 ± 0.5% in the heat condition and did not result in cognitive impairment. This results is supported by a previous study that showed the serial addition test, word recognition test, and trail making test were impaired at 2%~4% dehydration [28]. Another previous study reported that concentration and eye-hand coordination performance were impaired at 2% and 3% dehydration [29]. A review paper suggested that a dehydration level of 1% is a critical point of cognition impairment [30]. Collectively, these finding show that two simulated work activities in the heat did not cause severe hyperthermia and dehydration. Consequently, selective attention, namely SCWT and TMD were not impaired by heat stress. The SCWT was not affected by heat stress and/or two simulated activities. However, we observed TMD impairment at resting and TMD improvement following two simulated activities. Previous studies reported beneficial effects of acute bouts of exercise on mood state [31,32,33]. Another experimental study examined the effect of five exercise durations (10, 20, 30, 45, and 60 min) at three different recovery periods (5, 15, and 30 min) and concluded that acute exercise had positive effects on mood state, independent of exercise duration and recovery period [34]. BLA was significantly increased during B3, AE1, and AE2 in both conditions with a slight decrease following the 10-min recovery but did not differ between conditions. This result is in agreement with a previous study, indicating that intramuscular lactate concentration was not significantly changed in a hot environment compared to thermoneutral condition despite shorter exercise time to exhaustion and higher muscle and core temperature [35]. On the other hand, a more recent study by de Barros, et al. [36] observed higher BLA in the heat during steady state exercise. This discrepancy may possibly be attributed to the degree of heat stress, exercise intensity and fitness level (training status).

Previous studies have reported that exercise in a hot environment can induce greater oxygen debt and faster rate of BLA accumulation [37,38]. The exercise intensity at which an individual can clear blood lactate as quickly as lactate is accumulating is directly related to fitness level. For example, when a person is working at relatively lower intensity they are able to remove BLA before it accumulated because lactate can be circulated to other tissue and used as an energy source [39]. The adjusted exercise intensity during aerobic exercise could be responsible for a similar response in BLA.

The adjustment of exercise intensity for desired 75% of HRmax during aerobic exercise exhibits the similar responses in VO_2_ and MAP between conditions despite higher HR in a hot environment. During aerobic exercise in the heat, the treadmill incline and speed were decreased in the heat condition in order to maintain the target HR for all subjects. On the other hand, the anaerobic bout of exercise (lifting 22.7 kg sandbag) was not adjusted because it is a minimum requirement that first responders, specifically, EMT, should lift 22.7 kg repeatedly. Despite the compatible results, this study suggests that there may be an important implication that extended work in the heat would induce severe hyperthermia and dehydration such that cognitive performance becomes impaired.

This study has limitations that should be considered for generalization and interpretation. First, the subject population was limited to only a rural population of recent Indiana University of Pennsylvania EMT students. Therefore, future studies are needed to examine thermoregulatory, metabolism and cognitive performance in larger and diverse populations due to a growing portion of older adults and women in the workforce. Second, the simulated work activities in a hot environment were relatively short to detect adverse effects on physiological and psychological responses. Another limitation is methodological in nature, in that the CorTemp temperature sensor is accurate within 0.1 °C, which could lead to slightly altered core temperature recordings than are reported. Additionally, exercise intensity was reduced during the aerobic portion of protocol for the safety of subjects [40]. Therefore, future studies are needed to explore longer activities in a hot environment without exercise intensity adjustment. This would lead to greater increases in Tc and dehydration which would likely impair cognitive performance and mood sate.

## 5. Conclusions

This study has indicated that the magnitude of core temperature elevation and whole-body water loss were not different between conditions such that the Stroop color word test was not changed, over time, between conditions. Total Mood Disturbance was significantly impaired during resting in both conditions and was improved following two successive exercise bouts. There is a possibility that extended work in hot and humid environments may impose a psycho-physiological burden, and for this reason, prolonged work in this environment needs to be investigated.

## Figures and Tables

**Table 1:** Cognitive performance and subjective responses.

Variables	Condition	B1	B2	B3	AE1	AN1	AE2	AN2	R1
RPE	Neutral	6 ± 0	6 ± 0	6 ± 0	11 ± 1	11 ± 2	12 ± 1	12 ± 2	6 ± 0
	Heat	6 ± 0	7 ± 1	7 ± 1	12 ± 2	13 ± 2	14 ± 2 *	14 ± 3 *	6 ± 10
TS	Neutral	5 ± 1	5 ± 1	5 ± 1	7 ± 1	7 ± 1	7 ± 1	7 ± 1	5 ± 1
	Heat	6 ± 1	7 ± 1 *	7 ± 1 *	8 ± 0 *	8 ± 1 *	9 ± 2 *	9 ± 0 *	5 ± 1
Interference	Neutral	19.9 ± 8.7	20.1 ± 5.2	22.5 ± 6.7	-	-	-	-	22.9 ± 6.0
	Heat	20.1 ± 10.1	23.1 ± 8.3	24.3 ± 8.7	-	-	-	-	24.3 ± 4.2
TMD	Neutral	−95 ± 73	−95 ± 69	23 ± 25 ^a,b^	-	-	-	-	−98 ± 56 ^c^
	Heat	−85 ± 58	−75 ± 58	12 ± 65 ^a,b^	-	-	-	-	−66 ± 88 ^c^

Values are mean ± standard deviation (SD). B1, baseline; B2, second baseline; B3, third baseline, AE1, first aerobic exercise; AN1, first anaerobic exercise; AE2; second aerobic exercise; AN2, second anaerobic exercise; R1, recovery. RPE, rating of perceived exertion; TS, thermal sensation; Interference, interference score of Stroop color word test; TMD, total mood disturbance (higher score indicates poor mood state). ^a^ *p* ≤ 0.05 versus B1 at each condition, ^b^ *p* ≤ 0.05 versus B2 at each condition, ^c^ *p* ≤ 0.05 versus B3 at each condition, * *p* ≤ 0.05 versus neutral condition; -, Not applicable.
